# Lesions of the Trifacial (Especially Facial Neuralgia,) Resulting from Diseases of the Dental Organs and Adjacent Parts

**Published:** 1878-06

**Authors:** D. H. Goodwillie

**Affiliations:** New York City.


AMERICAN JOURNAL

OF

DENTAL SCIENCE.

Vol. XII. THIRD SERIES—JUNE, 1878. No. 2.

### ARTICLE I.

### *Lesions of the Trifacial (Especially Facial Neuralgia,) Resulting from Diseases of the Dental Organs and Adjacent Parts.*

D. H. GOODWILLIE, M. D., D. D. S., NEW YORK CITY.

[Read before the N. Y. Neurological Society.]

Much has been written on the physiology and pathology of this important nerve, and much remains to be learned. The object of this paper is to bring more prominently to the notice of the profession a great source of neuralgia of the head and face. Without going at length into preliminary anatomical and physiological details respecting this fifth nerve, which must be known or readily accessible to all, I will commence at once with the subject.

*Lesions of the Trifacial. (Facial Neuralgia.)*

The term neuralgia is from the Greek *neuron*, a nerve, and *algos*, pain. Neuralgia is a *condition*, or *effect*, and not a *cause*—and refers to pain of a paroxysmal character—localized or metastatic, without manifestation of any lesion at its seat. The pains are nearly always unilateral; and usually follow the course of particular sensory nerves. The character of these pains is mostly acute—intermittent—regular or irregular, according to the amount of irritation.

The pre-disposing cause of neuralgia may be of a general or local character; and to appreciate it fully, is to search until whatever lesion exists, is discovered.

I shall only allude to the central and reflex nervous diseases incident to first dentition, and will but direct your attention particularly to those more obseure affections of a neuralgic character dependent upon lesions of *second dentition.* These lesions naturally divide themselves into (1) those that are reflex—secondary or remote—and (2) those that are direct—immediate and from contiguity. In the former we may have epilepsy, neuralgia or paralysis; in the latter, local pain, facial palsy, or some forms of amaurosis. There are cases, however, which appear to comprise both conditions.

The branches of the trifacial appear to be the most susceptible to reflex action; and next to these, the cervical and brachial plexuses—hence, pains in the elbow, acromian process, insertion of the deltoid, neck or shoulder, with sometimes a loss of motor power.

Dr. Anstie, (Lancet, 1866, vol. II., pages 31, 32,) has mentioned two instances of wounds of the branches of the ulnar nerve causing reflex neuralgia of the fifth.

Reflex nervous irritations, from lesions of the dental organs, maxillary or nasal bones, are often very uncertain and capricious in their manifestations.

One may suffer much from a comparatively slight irritation, while in others the same condition will produce no result whatever.

There is undoubtedly a *neuralgic diathesis* which is hereditary, or induced, peculiarly in the centric or perhaps collateral relation of certain nerves. A *rheumatic* or *gouty diathesis* in one generation, may be followed in the next by a neuralgic diathesis; or it may pass over one generation to appear in the third. Pathological conditions of mind or body, may predispose to attacks of neuralgia. In great mental exercise, rather than in physical, does this so often occur in lesion of the trifacial from dental irritation.

All the following abnormities of the dental organs, maxillary or facial bones, have caused manifestations of reflex nervous irritation, namely :

Caries with or without exposure of the pulp-exostosis, hypertrophy of the cementum, osteo-dentine developments in the pulp cavity, periodontitis, periostitis, impaction of permanent teeth in the maxillary bones and maxillary abscess.

I will now proceed to illustrate by a narration of cases selected from very many that have come under observation.

CASE I.

*Neuralgia of the neck and arms, from exposed dental pulps, by caries.*

Mrs. A., of Philadelphia, came to consult me on account of a neuralgia for which she had been treated by her physician for the past two years without any permanent relief, and her suffering had steadily increased. She resorted hypnotics to obtain sleep. She complained of pain in her neck and arms, and the right side of her head. She said she was not aware that the teeth were ever the cause of such pain, and it had not occurred to her, nor had it been suggested to her by her medical attendant, that this might be the cause of her trouble. She was brought to me by a former patient who was " wiser in his generation " from having been a fellow sufferer. I made an examination of her mouth, and found two teeth on the right, and one on the left side of the mouth, with exposed dental pulps. The cavity in the enamel was very small, and she had not felt or even suspected that all her suffering came from her teeth. One of the dental pulps was on the point of suppuration, and had to be devitalized and the pulp extracted. The other two pulps were saved—irritation removed—inflamation subdued, then capped, and filled permanently with gold.

Three days after treatment was commenced, the neuralgia ceased, and has not since returned.

CASE II.

*Neuralgia from exposed dental pulps.*

Miss M. S., aged 16 years, was brought to me by her aunt, who said that her niece had suffered for the past two years with neuralgia of the face. It changed from one part of the face to another. Has had no appetite—was troubled in her sleep—was much depressed in mind, and weak in body. She had been compelled to give up her studies. As in the last case, I found exposed dental pulps, which were treated, and the neuralgia entirely disappeared. She is now in robust health.

CASE III.

*Neuralgia of the superior and inferior dental and infra-orbital nerves, from exposed dental pulp.*

E. W. D., aged 19 years, was sent to me by his family physician, November 25th, 1875, suffering from a neuralgia of the inferior and superior maxillary and infra-orbital nerves of the right side. His mother and grandmother were subject to facial neuralgia, and neuralgia in the feet. He has never before had neuralgia of the face in such a severe form. Has had his teeth cared for by one of our most skillful dentists—and has never had the slightest trouble with them. Has not had them examined for more than a year.

On examination, I found the jaws large and well developed, and all the teeth present except the right-lower wisdom tooth that was just now piercing the gum. The first superior bicuspid of this same side, had a large decay in it, and in consequence, the dental pulp was irritated. He would wake up at night with paroxysms of pain. I took away the irritant, and immediately his neuralgia stopped and has not returned.

CASE IV.

*Neuralgia from irritatiou of the dental pulp.*

In April, 1872, G. H. M., of New York, aged 36, an officer in the Western Union Telegraph Company, called on me respecting a neuralgia of the right side of his face, which at times gave him much trouble. He particularly noticed, he said, that after eating fruit, or any thing of an acid nature, it very much aggravated his suffering. He did not think his neuralgia came from his teeth as he had them well cared for, and never had any pain in them. Indeed, he had many sources from which he thought his neuralgia came. He had been under treatment but the neuralgia still persisted in annoying him.

On examining the mouth and throat, I found every thing in good order. There were no decays in the teeth; but on the upper molar of the right side, there was a very large amalgam filling, and immediately below, on a molar of the lower jaw, a large gold filling; as the mouth closed, these fillings touched each other. Suspecting that these teeth were the source of all his trouble, I applied a weak current of electricity to them and my suspicions were fully confirmed. The different kinds of metal in the fillings of these teeth, and the acid from the fruit, excited a galvanic action which was carried through the thin layer of dentine under these fillings, and over the dental pulp, and set up an irritation sufficient to have caused him all the neuralgia. To cure this, was only to stop the galvanic action on the dental filaments. The filling in both teeth was removed, and a very small amount of some non-conducting material placed in the bottom of each cavity, and over the region of the pulp to cut off all galvanic and thermal changes from the irritated pulp. Then both teeth were filled with gold and his cure was complete.

CASE V.

*Neuralgia from irritation of the dental pulp.*

I was called in by a surgeon of this city to see his wife who had been suffering for some time with neuralgia of the face and neck of right side. She had lost a number of her molars, and those remaining had to do double duty. All decayed teeth had been carefully filled with gold, and as they were small, no irritation was suspected to come from them. In one of the lower right molars, I discovered the cause of her neuralgia. By mastication the enamel had been worn off, and the irritation had been on the pulp by friction, and galvanic and thermal changes, together with a good deal of debility that she was suffering from. The local application of electricity to the pulp relieved the neuralgia almost instantly. Quinine was also administered. There has been no return of the trouble up to this time.

CASE VI.

*Neuralgia from irritated pulps.*

W. M. S., aged 48 years, from Virginia, R. R. Pres., sent to me by the President of this society, as he suspected that the neuralgia of his head proceeded from some irritation in the teeth or jaws since his case did not improve under his treatment. I found no decays in his teeth, but several of them had the enamel so worn by mastication that there was an irritation produced through the thin walls of dentine on the dental pulp.

Treatment, proper to such cases, relieved him entirely of his neuralgia, and " made a new man of him " as he expressed it.

CASE VII.

Hon. T. W. P., aged 57 years, has been a sufferer for some five years with facial neuralgia, and has had its foci at the temporal and supra-orbital points of both sides. He has been, since his youth, an inveterate chewer of tobacco. In several teeth the enamel was so much worn by mastication that the pulps were irritated, and in one or two teeth there was slight pulpitis.

The slightest friction, or thermal, or galvanic change, would bring on a paroxysm of neuralgia.

After the irritation and pulpitis was removed I put on gold crowns under which was placed some non-conducting material to prevent all thermal and galvanic action on the ulp.

CASE VIII.

*Neuralgia from deposits in the dental pulp.*

Mrs. C., aged 36 years, wife of a prominent surgeon, has been the subject of facial neuralgia for the past ten years.

Her husband consulted me in May, 1871, having suspected that possibly her neuralgia came from some of the dental organs. The principle focus was in the temporal, a point on the auriculo-temporal branch a little in front of the ear. It occasianally changed to the supra and infra-orbital nerves.

Mrs. C., has an undoubted neuralgic diathesis, as her family on her father's side for several generations back have been the subjects of neuralgia in some form. I found some teeth decayed, which I extracted; and, as I expected, found deposits in the pulp-chamber, and all along the canal. In some teeth I found the pulp-chamber filled with osteo-dentine granules, and the nerve alive half way up the canal. In others, the pulp-chamber and canal were half filled by deposits which crowded the vessels and nerve filaments into very small space, causing intense suffering.

She experienced great relief from the extraction of those teeth. It is important that all sources of irritation be taken away—such as metal fillings near the pulp, diseased gums, etc.

CASE IX.

*Neuralgia from osteo-dentine deposits in the dental pulp.*

H. G. H., aged 26 years, born in N. Y., very well developed, had severe neuralgia of the face but has never had toothache. On making an examination of the teeth, I found a superior molar decayed, and the pulp exposed. It was in such a condition that I considered it useless to try to save the pulp, and, therefore, put in a preparation to devitalize it. On his return next day, he expressed great delight in the relief he got from what he denominated a "tormenting pain."

On attempting to extract the devitalized pulp, I found the pulp chamber nearly filled up with a large deposit of osteo-dentine which required considerable cutting away of surrounding walls of the pulp chamber in order to remove it. There did not appear to be any deposit in the canal. After the decayed cavity had been filled with a non-conducting material until all signs of irritation might be expected to pass away, it was filled permanently with gold. A non-conductor was placed in the pulp chamber after the canal in the roots had been filled with gold, to separate it from the gold in the cavity of decay and thus prevent any galvanic or thermal action from being carried up the whole length of the tooth.

CASE X.

*Neuralgia from deposits in the pulp chamber.*

F. G., aged 41 years, sent to me by Prof. F. H. Hamilton, in June, 1870, has had neuralgia of the right side of the face for the past twenty years. It was contracted by exposure to wet and cold in the gold mines of California. It has gradually increased until now the paroxysms appear about every five minutes. He is well developed physically, and has always been healthy until the present trouble supervened. But he now presents a most woeful appearance from his long and constant suffering.

The pain has been somewhat changeable. At first it appeared in the inferior dental at its exit upon the face, at the mental foramen, and forward to the center of the chin. A section of this nerve was made twelve and seven years ago with only temporary relief.

He now suffers very much from the infra- and supra-orbital nerves,—the former, particularly. During his paroxysms of pain he has lachrymation and salivation ; and if in the middle of a sentence while speaking, he is obliged to stop short until it has passed away. He has taken all the known remedies for neuralgia ; yet it has steadily increased. He is willing to submit to any treatment to obtain relief from his great trouble. No history was given of hereditary neuralgia rheumatism or gout.

The mouth was examined and all the teeth remained except a few of the back molars. The remaining teeth were all sound with the exception of a very small decay in two superior molars which were filled and could cause no trouble. From my past experience in these cases, I was led to suspect that there were deposits in the dental pulps. A few teeth were extracted, and when the teeth were split opon, osteo-dentine deposits were found enclosed in the vessels of the pulp. All the remaining teeth were extracted ; and a section being made deposits were found either in the pulp chamber or in the nerve canal. In some of these teeth presented, will be seen large and small osteo-dentine granulus in the substance of the pulp. In the canal of the roots will be seen also a narrowing of the canal by deposits to the walls.

As these calcified masses formed, the vessels of the pulp were encroached upon, and strangulated, and the pressure on the nerves caused severe pain.

In some of the teeth it will be seen that the dental pulp was entirely cut off in the fangs by deposit in the canals.

This patient remained under observation for some time before he returned to his home and up to that time appeared entirely relieved from his suffering, since which time, I have not heard from him. I cannot say that the prognosis will be entirely favorable in this case.

It being of long standing—the patient0apparently in the decline of life—pathological changes in the structure of the nerve tissue may have taken place. The lachrymation and salivation is an unfavorable feature in the case, as it shows that he is suffering from secondary affection of the glands.

Whether these deposits in the tissue of the pulp are the true cause of his neuralgia, or a consequent in this case, is difficult to decide. Certain it is, that it has been a source of intense suffering.

CASE XI.

*Catalepsy from polypoid growth of the pulp.*

In 1870, a physician brought his son, aged 14 years, to me, and stated that he had a cataleptic fit the day before, and that in investigating the cause he observed a growth in one of his lower molars. Thinking this to be very unusual, he consulted me concerning it. On examination, I found this to be a polypoid growth from the pulp. The tooth was very much decayed, and the growth more than filled the cavity of the tooth; for on closing the mouth the upper tooth pressed upon it and gave rise to such an amount of irritation as to cause the trouble.

I took out the tooth and he has not had any return of the catalepsy since.

These polypoid growths appear on their more superficial portions to consist of cells about 1-2000 of an inch in diameter. Deeper in the substance of polypus, it is more of a fibrous nature, and contains many capillary blood vessls.

Sometimes the surface is found to to be indistinctly covered with epithelium. Occasionally the surface appears like true gum.

CASE XII.

*Neuralgia of the infra- and supra-orbital and optic nerves from hypertrophy of the cementum and abscess.*

Rev. Dr. J. D. W., came to see me in October, 1872, respecting a neuralgia and aphonia from which he was suffering, which greatly interfered with his duties. He describes his pain as a wave that commenced on his cheek and the sides of his nose (infra-orbital;) then it passes to his eyes, producing dimness of vision; and then by the supra-orbital it passes over the forehead to a point on the frontal eminence at the coronary suture. On examining the mouth I saw that the soft palate on the right side was very red and swollen, and that it passed down the palato-pharyngeus muscle. A laryngoscopic examination revealed redness and thickness of right arytenoids and vocal bands, and slight paralysis of the same side—a rhinoscopic examination could not well be made, on account of the thickness of the soft palate. He had some naso-pharyngeal catarrh. I suspected the cause of the neuralgia was the right superior wisdom tooth, and on extracting it found a large abscess at its root, with hypertrophy of the cementum.

After a short treatment, his neuralgia and aphonia disappeared.

November 26, 1875, the doctor came again suffering from his neuralgia, but without the pharyngeal difficulty.

He had only four molars remaining in the upper jaw. These were extracted and the roots found to be very much hypertrophied, and the nerves and vessels entirely shut off by the deposit in two teeth, and a third nearly closed. The peridental membrane was very hyperæmic. A portion of the external alveolus came away, being adherent to the roots.

December 29, 1875, the doctor writes me that his neuralgia has all disappeared.

CASE XIII.

*Neuralgia of inferior and superior dental nerves, from a bristle in the pulp canal.*

Miss A. N. complained of a severe neuralgia of the inferior and superior dental, and infra-orbital nerves of the left side, which came on quite suddenly while at dinner; and has since given her no rest. Nervines were administered by her family physician, but to no purpose; and she had been advised to try a change of climate. She was about to carry out this advice, when she was brought to me. Up to the time that her neuralgia commenced, she had enjoyed excellent health. I could not discover any predisposing cause for her severe trouble, and therefore considered that it proceeded from a local irritant. Her teeth had been well cared for, and she had never had any trouble from them. After a very careful examination, I discovered a small fistulous opening behind an upper molar tooth, and the probe passed down to a root of the tooth. There was no inflammation about it; nevertheless I advised her to have it taken out. On extracting it, I found, sticking out of the canal, at the apex of the root, a bristle which had probably been disengaged from her tooth brush and found its way into the canal, and during mastication had been driven through the end of the root into the nerve. There was slight necrosis at the apex. It is needless to say that her neuralgia vanished instantly.

The following cases show that irritation of the spine may cause reflex action of some branches of the trifacial.

### CASE XIV.

*Reflex action from spinal disease.*

Minnie M., aged 14 years, suffering from disease of the spine, for which she has received treatment.

At present one of the most uncomfortable features of her case, and that one which gives her parents some anxiety, is the terrible grinding of her teeth in her sleep. Her mother says that she rises, some nights, several times to go to the adjoining room and awake her.

The teeth and mouth are in a healthy condition. Her front teeth are worn off very much by constant grinding, and the pulps would soon have been reached had not a splint of gold and hard rubber been made to cover over the crowns of the teeth, to be worn during her sleeping hours. Her spinal trouble as well as the reflex difficulty, are now very much improved.

### CASE XV.

A young lady applied for relief from a neuralgia that affected her jaws and the lower part of her face.

From her history, I could not make out any hereditary tendency to neuralgia, neither could I discover any local lesion from the dental organs, or maxillary bones. Thinking her trouble was perhaps due to some mal-assimilation of food by some perturbation of the fluids, I at once tasted the saliva, and found it quite acid.

On questioning her respecting her digestion, she said that her stomach had given her some distress; but since the neuralgia set in it was not so bad. She then told me that she was very fond of candy, and was never without it. The cause of her neuralgia was soon solved. The acid condition by the fermentation of the sugar in the fluids of the mouth and stomach, produced an irritating effect on the dental nerve filaments, thus giving rise to her neuralgic trouble. The eating of acid or fruits will sometimes produce the same effect. An alkaline condition will also do the same. I forbade the use of candy, advised plain food with plenty of out-door exercise, and her neuralgia soon left her.

CASE XVI.

*Neuralgia from maxillary abscess.*

A gentleman was brought to me by his family physician, complaining of severe neuralgia in the left ear, with partial deafness, of the inferior maxillary nerve at its exit from the mental foramen and forward to symphysis and border of the lip; and occasional twinges in other nerves of that side. Has been suffering from this neuralgia for two years. His habits were regular, and he had always enjoyed good health up to this time. He was an inveterate chewer of tobacco, and his neuralgia had increased that habit, and it consequently had no good effect on his trouble.

Rhinoscopic examination revealed nothing special. Anterior and posterior nares healthy with the exception of some thickening of the mucous membrane. Jaws very well developed and all the 32 teeth were present without decay, as I made a most thorough examination of them with a very delicate instrument.

Placing my patient in a very strong sun light, I discovered that the wisdom tooth of the left lower side was a shade darker than the rest, and worn somewhat on the crown by mastication. Electricity indicated that this tooth was the cause of some trouble. I next cut through the enamel where it had been nearly worn off by mastication, but found no sensation under the enamel and on the surface of the dentine where it would be natural to find it in healthy teeth. Concluding the dental pulp was dead, I drilled into it and found this to be the case—pus coming out after my instrument.

The tooth was extracted and a pus sac as large as the tooth itself came out hanging on the fangs. Below this sac, in the jaw, there was some necrosis of the cancellated structure of the bone.

The joy of my patient was very great on being so speedily and perfectly relieved of a long and painful neuralgia.

CASE XVII.

*Neuralgia from abscess in the antrum of Highmore.*

I was called to see Mrs. A. H., aged 44 years, of Philadelphia, who was suffering from a neuralgia of the right infra-orbital, superior and inferior maxillary nerves. There was some dimness of vision of the right eye and slight iritis.

A year before she had some roots extracted from the upper jaw of that side, from which she obtained relief from tooth pain. Shortly after her neuralgic trouble commenced, which steadily increased notwithstanding all treatment. During this time, she had all her teeth extracted, but the neuralgia was no better. When I saw her the alveolus appeared healthy, except that on the right side there was some bulging of the buccal wall of the antrum, and also in the palate on that side.

I also discovered a very small sinus through the alveolar ridge leading towards the antrum, which admitted only the smaller probe. It did not pass into the antrum. There was some obstruction.

There had been so small a discharge from this sinus that the patient was not aware of its existence.

Recognizing her trouble as connected with the antrum, I trephined through the track of the sinus and found the apex of an exostosed root of a tooth that had most probably been pushed into the antrum by the efforts to extract it.

After some months of local and general tonic treatment, she entirely recovered.

